# Leptospirosis: A Rare Cause of Hyperbilirubinemia and Acute Kidney Injury in a Non-endemic Area

**DOI:** 10.7759/cureus.30963

**Published:** 2022-11-01

**Authors:** Marko Kozyk, Kateryna Strubchevska, Brian Nadeau

**Affiliations:** 1 Internal Medicine, Beaumont Hospital, Royal Oak, USA; 2 Gastroenterology and Hepatology, Beaumont Hospital, Royal Oak, USA

**Keywords:** leptospires, icterus, acute kidney injury, hyperbilirubinemia, leptospirosis

## Abstract

Leptospirosis is a zoonosis with clinical manifestations caused by a pathogenic bacterium of the genus *Leptospira*. A high index of suspicion is required to make the diagnosis. The unusual constellation of symptoms makes the diagnosis of leptospirosis challenging and requires a comprehensive approach while treating the patient. This case illustrates the potential for severe complications of untreated leptospirosis and underlines the importance of a holistic approach to the diagnosis and treatment of patients with perplexing clinical presentations.

## Introduction

Leptospirosis is a zoonosis with variable clinical manifestations caused by a pathogenic bacterium of the genus *Leptospira*. It occurs in both temperate and tropical regions. A systematic review and modeling exercise estimated that there were 1.03 million cases worldwide annually, with 58,900 deaths. Leptospirosis stays among the leading zoonotic causes of morbidity worldwide and accounts for numbers of deaths, which approach or exceed those for other causes of hemorrhagic fever. The highest morbidity and mortality were estimated to occur in resource-poor countries, which include regions where the burden of leptospirosis has been underappreciated [1]. The clinical course of leptospirosis is protean; most cases are self-limited or subclinical while some are severe and potentially fatal. The illness generally presents with the abrupt onset of fever, rigors, myalgias, and headache in 75% to 100% of patients following an incubation period of 2 to 26 days (average 10 days). Leptospirosis may be complicated by jaundice and renal failure ("Weil's disease"), pulmonary hemorrhage, acute respiratory distress syndrome (ARDS), uveitis, optic neuritis, peripheral neuropathy, myocarditis, and rhabdomyolysis [2]. Here, we present a case of leptospirosis that was remarkable for its acutely worsening clinical course and vague presentation.

## Case presentation

A 39-year-old female, residing in Michigan, USA, with no significant medical history presented to the emergency department for evaluation of sharp pain in the right lower back and buttock, radiating down the back of her right leg. She was diagnosed with sciatica and treated with a course of prednisone and methocarbamol. She was then discharged from the hospital. The patient was readmitted four days later with complaints of fatigue, myalgias, nausea, vomiting, diarrhea, and yellowing of the eyes. She denied any recent travel or sick contacts. The patient reported consuming beef tacos from a local restaurant for the last three days. Her vitals on admission revealed a temperature of 100.2 °F (37.9°C), blood pressure of 137/85 mm Hg, and a heart rate of 99 beats/min. Laboratory tests were significant for leukocytosis (white blood cells: 21.9 × 109/L), anemia (hemoglobin: 9.6 g/dL), thrombocytopenia (platelets: 42 × 109/L), haptoglobin of 308 (reference range, 40-250) mg/dL, elevated aspartate aminotransferase (AST) and alanine transaminase (ALT) to 100 U/L, total bilirubin of 13.2 mg/dl (direct bilirubin: 9.4 mg/dl), and creatinine of 5.05 mg/dl. Urinalysis was positive for bilirubin, urobilinogen of 4.0 mg/dL, 2+ blood, protein of 500 mg/dL, and WBC of 11-20/hpf. The patient had diuresis and her average daily urine output was 1450 ml since the presentation. Ultrasound of the abdomen, kidneys, and bladder was unremarkable. Sonographic Murphy’s sign was negative and the biliary system was non-dilated. Given her fever, anemia, and thrombocytopenia, thrombotic thrombocytopenic purpura was suspected and eventually ruled out with a negative schistocyte smear. Anti-neutrophil cytoplasmic antibody (ANCA)-associated vasculitis and systemic lupus erythematosus were also suspected but eventually excluded due to her negative serologic workup and lack of clinical criteria. Extensive infectious workup was negative for viral hepatitis, coronavirus disease 2019 (COVID-19), Cytomegalovirus (CMV), influenza, respiratory syncytial virus (RSV), human immunodeficiency virus (HIV), and Epstein-Barr virus (EBV). However, leptospira immunoglobulin M (IgM) antibodies were positive by enzyme-linked immunosorbent assay. Based on high clinical suspicion and a positive serologic test, the patient was started on oral doxycycline 100 mg twice a day. The patient was discharged from the hospital in a week with a creatinine level of 1.18 mg/dL and significant clinical improvement.

## Discussion

Leptospirosis is an infection distributed worldwide, with most clinical cases occurring in tropical areas. In the United States, Hawaii consistently reports the most cases [3]. The number of reported cases in the Midwestern USA is low, but unfortunately, may more often be missed. There is a need for a comprehensive approach when suspecting the disease. Based on a Detroit study, there was a correlation between the degree of rat infestation and seropositivity rates. Approximately 30% of children in urban Detroit demonstrated serologic evidence of previous leptospirosis infections [4].

Human infection usually results from exposure to environmental sources such as animal urine, contaminated water, soil, or infected animal tissue. In our case, leptospirosis acquisition was likely related to the ingestion of food contaminated with urine. The first step in the pathogenesis of leptospirosis is the penetration of tissue barriers. The second step in pathogenesis is hematogenous dissemination when pathogenic leptospires make their way into the bloodstream and persist there during the leptospirosis phase of the illness [5].

Clinical manifestations of the disease can include fever, rigors, myalgias, and headache in 75% to 100% of patients [2]. However, sometimes, presentation is atypical, and the disease can manifest as back or leg pain. A tip-off to the identification of leptospirosis is conjunctival suffusion (dilatation of conjunctival vessels without purulent exudate), which occurs frequently in leptospirosis but is uncommon in other infectious diseases. Additional ocular findings typically include subconjunctival hemorrhage and icterus [5].

Aseptic meningitis is observed in 50% to 85% of patients if cerebrospinal fluid (CSF) is examined after seven days of illness [6]. Even though most cases of leptospirosis are mild to moderate, leptospirosis may be complicated by jaundice and renal failure, pulmonary hemorrhage, and acute respiratory distress syndrome (ARDS) [7]. Renal failure is often non-oliguric and associated with hypokalemia. Proteinuria, pyuria, granular casts, and occasionally microscopic hematuria are commonly seen on urinalysis. The pathogenesis of acute kidney injury is perplexing due to the direct nephrotoxic action of *Leptospira interrogans*, hyperbilirubinemia, rhabdomyolysis, and hypovolemia. Histological findings include interstitial nephritis and acute tubular necrosis. Supportive renal replacement therapy may be required during the acute phase, although renal function recovery is fast and complete after six months [8].

The diagnosis is made most frequently by serologic testing although molecular diagnostics have utility and are increasingly available. The microscopic agglutination test is considered a gold standard test in diagnosing leptospirosis; however, IgM enzyme-linked immunosorbent assay is the most widely used. In the absence of a definitive laboratory diagnosis, the administration of empiric treatment is also considered appropriate [2].

Most patients with leptospirosis improve spontaneously even without treatment. However, antibiotics shorten the duration of illness and reduce the spread of the infection. Therefore, treatment with oral doxycycline or oral azithromycin is usually chosen.

**Figure 1:**
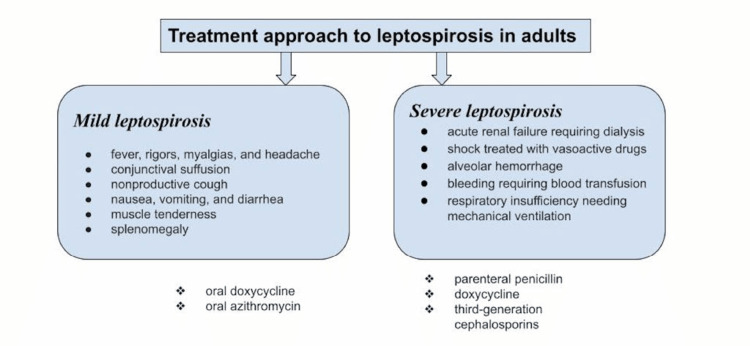
Treatment approaches to leptospirosis in adults

Figure 1 shows the treatment approach to leptospirosis in adults.

Patients who have severe leptospirosis are treated with parenteral penicillin, doxycycline, and third-generation cephalosporins [3]. There is no human vaccine widely available. Avoiding potential sources of infection, administration of prophylaxis for individuals at high risk of exposure, and animal vaccination are all acceptable options for prevention [8].

## Conclusions

Leptospirosis has a wide spectrum of clinical symptoms, including liver and kidney involvement, which represent life-threatening manifestations of the disease. Therefore, clinicians should have a high index of suspicion to make a prompt diagnosis and initiate therapy.
